# Urban life promotes delayed dispersal and family living in a non-social bird species

**DOI:** 10.1038/s41598-020-80344-8

**Published:** 2021-01-08

**Authors:** Álvaro Luna, Nicolás A. Lois, Sol Rodríguez-Martinez, Antonio Palma, Ana Sanz-Aguilar, José L. Tella, Martina Carrete

**Affiliations:** 1 Department of Conservation Biology, Estación Biológica de Doñana - CSIC, Sevilla, Spain; 2 Laboratorio de Ecología y Comportamiento Animal. Departamento de Ecología, Genética y Evolución, Universidad de Buenos Aires, Buenos Aires, Argentina; 3 Instituto de Ecología, Genética y Evolución de Buenos Aires, Consejo Nacional de Investigaciones Científicas y Técnicas, Buenos Aires, Argentina; 4 Department of Evolutionary Ecology, Estación Biológica de Doñana - CSIC, Sevilla, Spain; 5 Animal Demography and Ecology Unit, IMEDEA (CSIC-UIB), Esporles, Spain; 6 Applied Zoology and Conservation Group, University of Balearic Islands, Palma, Spain; 7 Department of Physical, Chemical and Natural Systems, Universidad Pablo de Olavide, Sevilla, Spain

**Keywords:** Evolutionary ecology, Ecology, Behavioural ecology

## Abstract

In some vertebrate species, family units are typically formed when sexually mature individuals delay dispersal and independent breeding to remain as subordinates in a breeding group. This behaviour has been intensively studied in gregarious species but has also been described in non-social species where ecological and evolutionary drivers are less known. Here, we explore factors that favour delayed dispersal and family living and potential benefits associated with this strategy in a non-social, monogamous species (the burrowing owl, *Athene cunicularia*) occupying urban and rural habitats. Our results show that family units arise when first-year individuals, mainly males, delay their dispersal to stay in their natal nests with their parents. This delayed dispersal, while still uncommon, was more prevalent in urban (7%) than in rural (3%) habitats, and in areas with high conspecific density and productivity. Birds delaying dispersal contributed to the genetic pool of the offspring in 25% of the families analysed, but did not increase the productivity of the nests where they remained. However, their presence was related to an improvement in the body condition of chicks, which was ultimately linked to a slightly positive effect in offspring future survival probabilities. Finally, delayed dispersers were recruited as breeders in high-quality urban territories and closer to their natal nests than individuals dispersing during their first year of life. Thus, our results suggest that delaying dispersal may be mainly related to opportunities to inheriting a good quality territory, especially for males. Our study contributes to understanding the role played by habitat quality in promoting delayed dispersal and family living, not only in social but also non-social species, highlighting its impact in the ecology and evolution of animal populations.

## Introduction

In their route to breeding, individuals within a species can differ in their dispersal decisions^1–4^, some searching for a vacancy at different distances from their birth area (i.e., disperser) and others remaining in the surroundings (i.e., philopatric). Dispersal may confer fitness advantages by reducing potential costs of kin competition and inbreeding, allowing individuals to leave their natal areas and facilitating species range expansions^5–8^. Conversely, philopatry may provide advantages through familiarity with the natal environment and conspecifics and may also promote kin cooperation^7,9,10^. However, natal philopatry can also entail disadvantages by constraining habitat selection options and limiting the escape from ecological traps^11–13^. Thus, natal dispersal is an important life-history trait that may influence an individual’s prospects as well as species ecology and evolution, including the distribution, dynamics, persistence, and genetic composition of populations^14–19^.

In some vertebrate species, however, sexually mature individuals may delay dispersal and independent breeding to remain as subordinates in a group, forming families (i.e., parent–offspring associations that go beyond the period when offspring are actively provisioned by their parents^20^) and even cooperative units (i.e., offspring remaining with their parents beyond nutritional independence and helping them in subsequent breeding^20–22^). Given that evolutionary theory predicts that individuals should maximise their fitness, several studies have attempted to understand why some individuals choose to delay dispersal. The “ecological constraints” hypothesis poses that individuals delay dispersal when suitable breeding vacancies are limited (e.g., due to habitat saturation) or too costly to find^11,23,24^ or when differences between breeding sites are very marked (i.e., presence of high and low-quality territories^25^). Nevertheless, many species facing those constraints disperse after independence, whereas offspring in several species delay dispersal in unsaturated habitats^26,27^, suggesting that other factors should be involved in explaining delayed dispersal. The “benefits of philopatry” hypothesis predicts that individuals intentionally delay dispersal when the fitness benefits (e.g., learning, antipredator protection, or food provisioning) of remaining as a subordinate in a territory (in terms of survival and/or reproductive output) exceed those of leaving^17,28,29^. A factor now recognized as crucial to allow individuals to delay their dispersal is parental facilitation (the adaptive delayed dispersal hypothesis^30^), as a prolonged investment in offspring beyond their independence can be adaptive in some circumstances (long-lived species) but too costly to afford in others (shorter-lived ones^31^). Thus, life-history traits may predispose certain species toward family living, although its expression can highly depend on ecological conditions and social factors faced by each population^27,32^. Focussing research on non-social species that do not perform any group activity^25,33^ and comparing populations where individuals face different ecological pressures can provide novel insights on the drivers maintaining or even promoting delayed dispersal and, therefore, family living.

Human activities can dramatically change ecological conditions^34,35^, exposing animals to situations they have not experienced in their evolutionary history, and leading some once-adaptive behaviours to become maladaptive^36^. Urbanisation, in particular, is the most drastic and persistent human-driven alteration of the landscape, which creates new habitats starkly different from the natural areas it replaces^37,38^. Although urbanisation leads to an overall loss of biodiversity (the so-called ‘biotic homogenisation process’^37,39,40^), some species seem to prosper in these environments^41^, taking advantage of low predation pressure^42^ and high food availability and predictability^43^. Larger population densities and higher reproductive parameters in urban than in rural habitats have been recorded in different city-dweller species^41,44–46^, suggesting a significant role for predation release in their success^47,48^. However, although these species may take advantage of the new environment, they may also face new ecological conditions and selection pressures that can induce changes in behavioural^41,49–52^ and reproductive strategies^36,53,54^. These changes may translate into differences in dispersal patterns^55,56^ and even population structuring^57–59^.

Here, we investigate drivers of delayed dispersal in the burrowing owl, *Athene cunicularia*, a non-social, monogamous species^36^ intensively studied as a model to understand drivers and consequences of urban life. Previous research has shown that breeding densities and productivity of this species are notably higher in the city than in their adjacent rural areas due to predation release^48^. This fact, in addition to the differential behavioural profiles of urban compared to rural birds^41,52,60^, has promoted differences in the natal and breeding dispersal patterns of both groups of birds, with urban individuals being less prone to disperse or dispersing at shorter distances than rural ones^55,56^. However, it is unknown whether urban life and their associated changes in an individual’s natal dispersal pattern could also affect the decision of some to remain in their natal nests until their second year of life, delaying dispersal and leading to the formation of unusual family units. Therefore, our aims in this study were: 1) to ascertain the role played by delayed dispersal as a driver of family living in a non-social, monogamous species occupying urban and rural habitats, 2) to explore factors favouring the appearance of these family units, and 3) to assess potential consequences for individuals delaying dispersal and their associated breeders.

## Results

### Delayed dispersal as a route to family living

During the study period, we monitored 5776 breeding events, 4.62% of which corresponded to breeding units (n = 267) formed by more than two adults (hereafter, family groups). Most of these family groups (97%) included only one additional adult male, while in 3% of the cases we recorded 2 additional adults (two males, one male and one female, or two females). Ringing data show that extra-individuals in these family units were mainly offspring of the main breeders born in the previous breeding season, both when the social identity of all individuals was known as well as when only one of the breeders was of known identity (n = 22 cases; Table S1). Genetic parentage analysis (Cervus and relatedness analysis) consistently assigned 19 out of 23 chicks born in territories where at least the two adult males of the family unit were genotyped to one of the potential fathers (Table S1). The four offspring with inconsistencies between Cervus and relatedness results correspond to a nest where a likely extra-pair fertilisation took place and another territory with high inbreeding (mother and putative father with a parent–offspring kin relationship), which hinders genetic assignment. In one out of the eight families, the male delaying dispersal was the father of the offspring (Table S1). We also found high relatedness values between both males and among the three adult individuals in family units (Table S1), indicating siblings or a parent–offspring relationship between them. Altogether, these results suggest that the extra adults present in the family units were offspring of the other two breeders that remained in their natal nests but rarely contribute genetically to the brood. Thus, from now on, we categorized nests as having been owned by a breeding pair when only two adult individuals (one male and one female) were systematically observed; otherwise, a family unit was formed by the main breeding pair (one male and one female) plus one (or sometimes two) extra adult individual(s).

**Figure 1:**
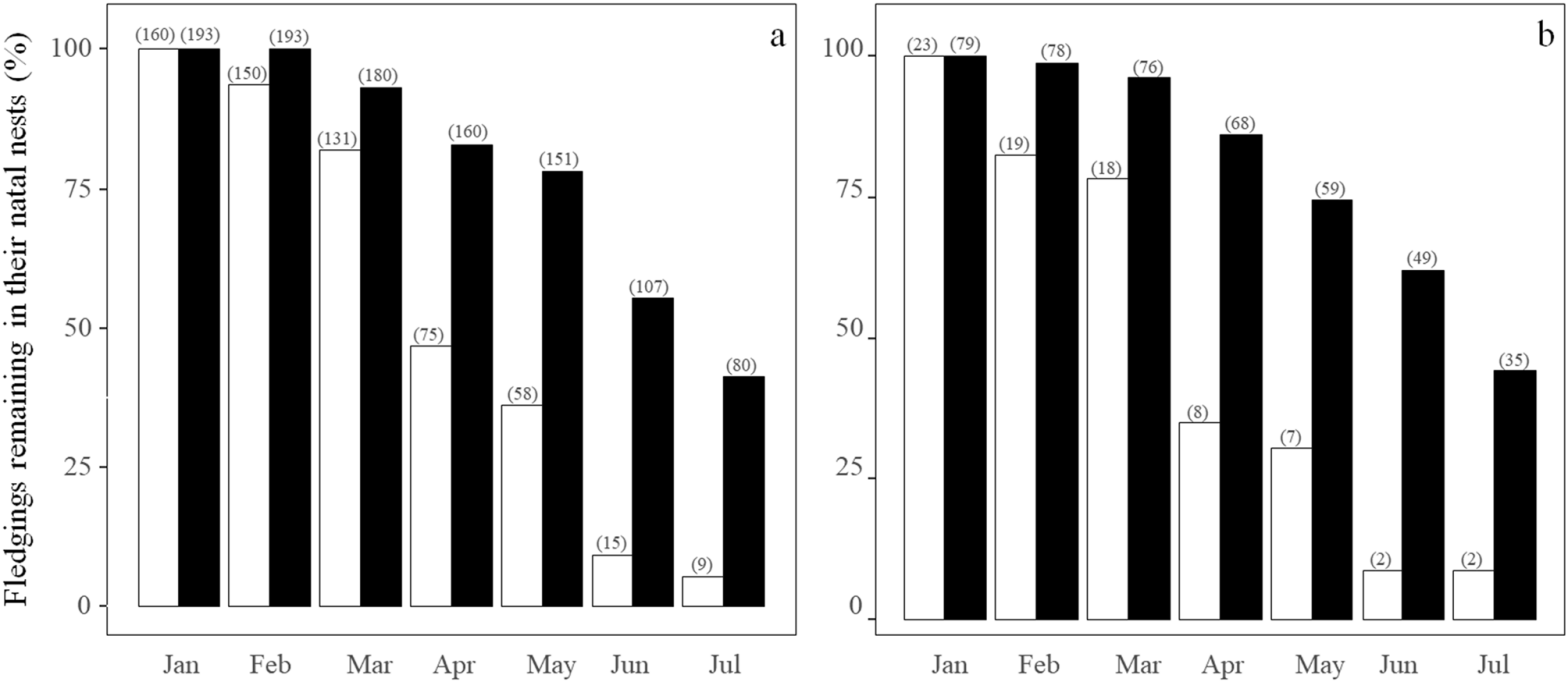
Percentage of fledglings remaining in their natal nests beyond the period when they are actively provisioned by their parents in urban (black bars) and rural (white bars) areas. (**a**) All individuals monitored; (**b**) ringed birds monitored. In brackets, number of individuals observed per month.

Our monthly observations performed in 2010 confirmed that a large proportion of chicks stay in their natal nests long beyond the period when they are provisioned by their parents (Fig. 1). The proportion of monitored birds that remained in their natal nests was higher in urban than in rural habitats (estimate for urban habitats: 1.37, 95% CI: 0.39—2.35), and decreased with time (estimate: -0.73; 95% CI: -0.88—-0.58), more markedly in rural than in urban areas (interaction habitat*time: 0.16, 95% CI: -0.05—0.38). Similar results were obtained when our sample was restricted to birds ringed as chicks and whose natal nests were assigned without doubts (estimate for urban habitats: 1.73, 95% CI: 1.05—2.42; time: -0.61, 95% CI: -0.79—-0.44). Model fits can be checked in the Supplementary Material (Fig. S1a,b, respectively).

### Factors driving delayed dispersal and family living

**Figure 2:**
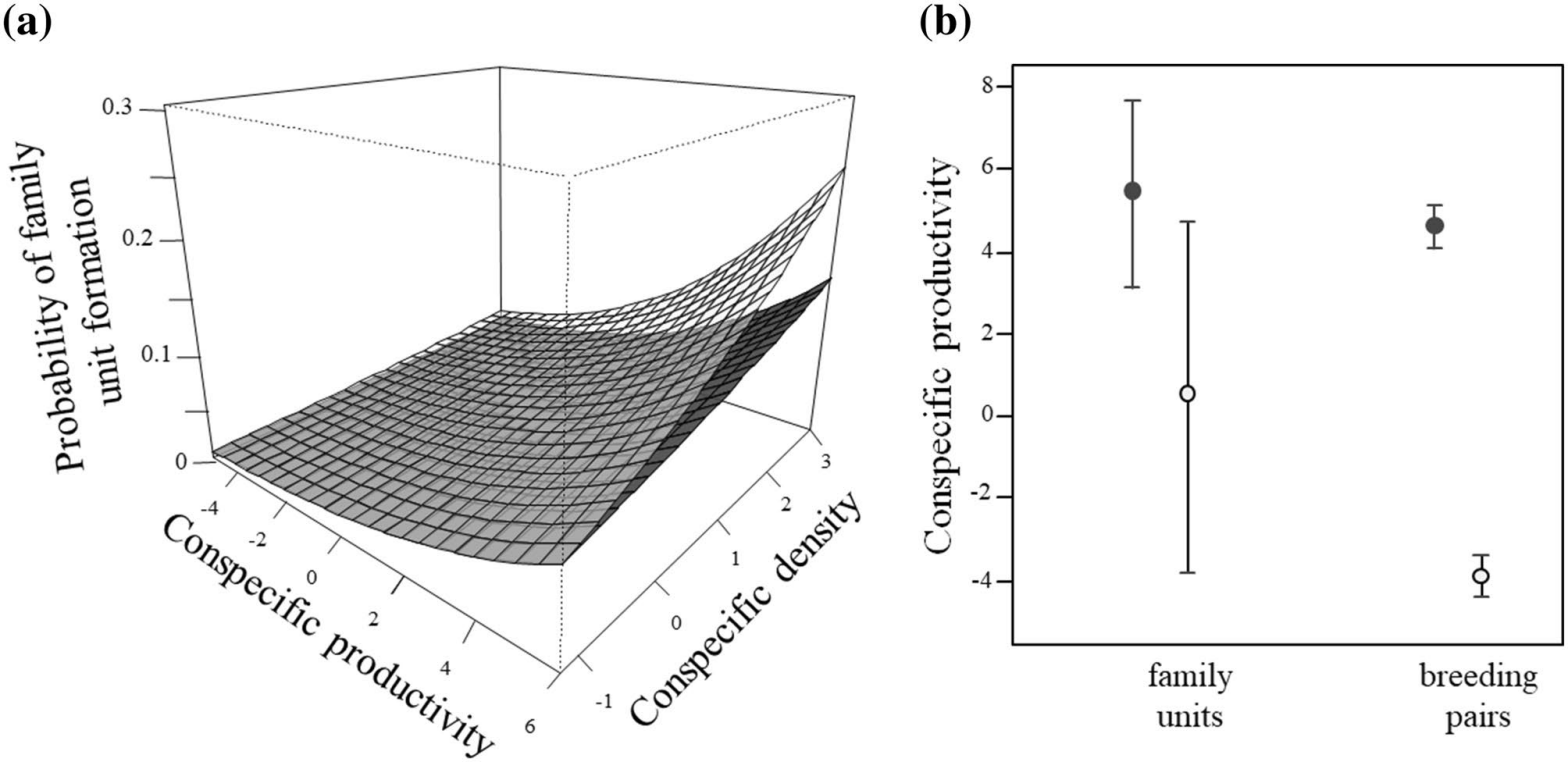
(**a**) Relationship between conspecific density and productivity and the probability of urban (dark grey) and rural (pale grey) burrowing owls *Athene cunicularia* to form family units through delayed dispersal. (**b**) Differences in conspecific productivity in the surrounding or urban (black dots) and rural (white dots) family units and breeding pairs.

Based on previous results, we assumed that most family units present in our study populations were formed because an individual delayed its dispersal to stay in its natal nest until the next breeding season. These family units were more frequent in urban (7%, n = 2574 breeding events) than in rural (3%, n = 3202 breeding events) areas, and where conspecific density and productivity were higher (Table 1, Fig. 2a). Differences in conspecific productivity between family units and breeding pairs were more marked in rural than urban areas (Fig. 2b). However, the estimate for the interaction between conspecific productivity and habitat barely overlapped zero (Table 1; see model fit in Fig. S2). Models explained less than 5% of the total variability in the data, suggesting that our ability to predict the formation of family units in this non-social species is very low.

**Table 1:** Models obtained to assess the effects of habitat, conspecific density, and productivity on the probability of a breeding site being occupied by a family unit of burrowing owls *Athene cunicularia*.

Model selection					Model averaging			
Model	k	AICc	∆AICc	W	Variable	Estimate	2.5% CI	97.5% CI
Habitat + conspecific productivity + conspecific density	5	2067.71	0.00	0.29	**Conspecific productivity**	**0.22**	**0.06**	**0.38**
Habitat + conspecific productivity + conspecific density	5	2067.71	0.00	0.29	**Habitat(urban)**	**0.53**	**0.22**	**0.85**
Habitat*conspecific productivity + conspecific density	6	2067.95	0.24	0.25	**Conspecific density**	**0.30**	**0.13**	**0.47**
Habitat*conspecific density + conspecific productivity	6	2068.77	1.06	0.17	Habitat (urban)*conspecific productivity	−0.16	−0.39	0.07
Habitat + conspecific density	4	2075.85	8.14	0.01	Habitat (urban)*conspecific density	−0.14	−0.41	0.14
Habitat*conspecific density	5	2076.32	8.61	0.00				
Habitat*conspecific productivity	5	2079.56	11.86	0.00				
Habitat + conspecific productivity	4	2080.30	12.60	0.00				
Habitat	3	2084.95	17.24	0.00				
Conspecific density	3	2093.99	26.29	0.00				
Conspecific productivity	3	2116.54	48.83	0.00				
Null	2	2135.06	67.35	0.00				

Estimates and 95% confidence intervals (2.5% and 97.5%) were assessed after model averaging. All models were run including year as a random term. k: number of parameters, AICc: Akaike Information Criterion corrected for small sample sizes, ΔAICc: difference between the AICc of model i and that of the best model (i.e. the model with the lowest AICc), w: Akaike weights. The fit of the model including all variables used in model averaging can be checked in Fig. S2. In bold, variables receiving strong support (i.e., the 95% confidence interval did not overlap with zero).

**Figure 3:**
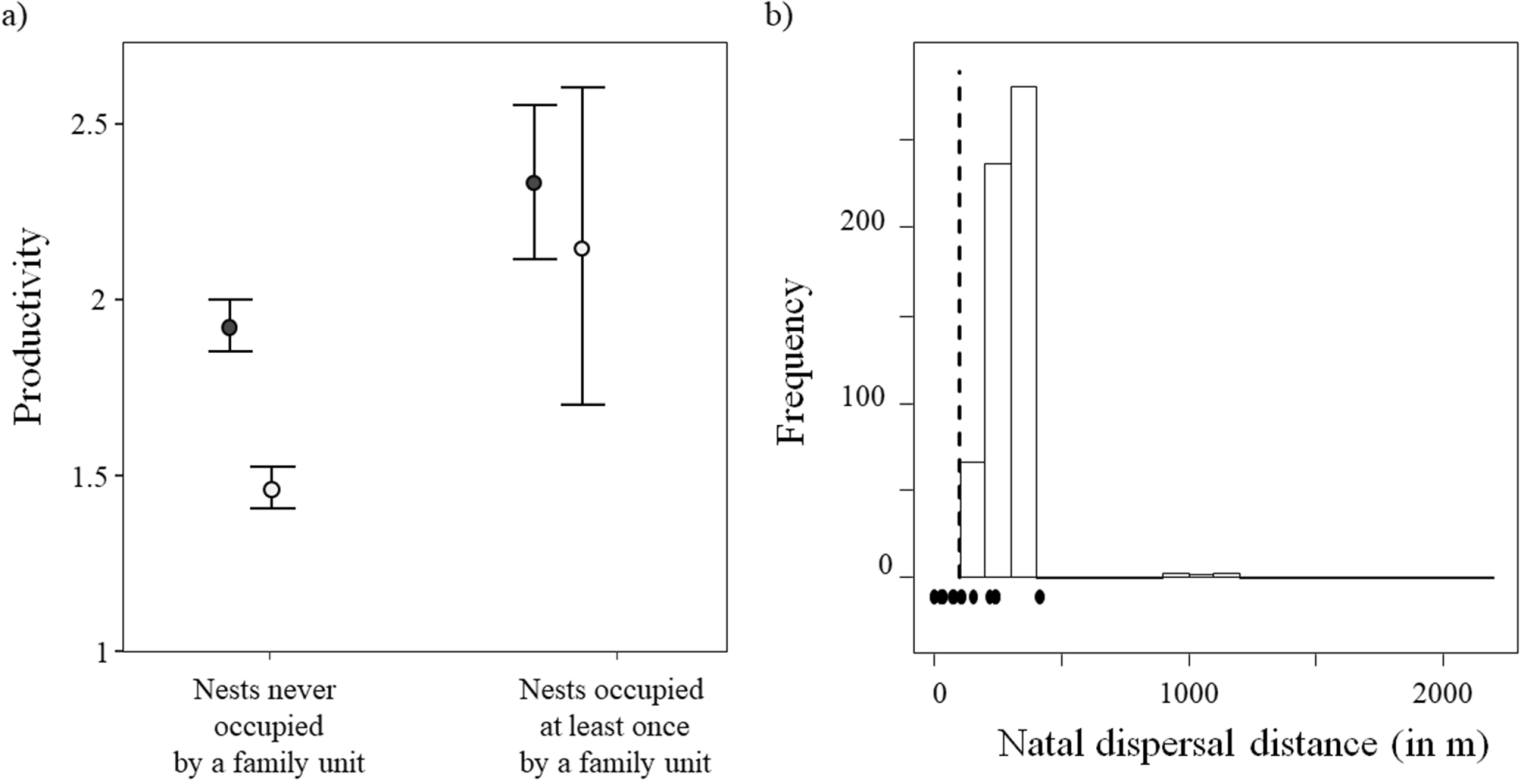
(**a**) Annual productivity (mean ± 95% CI) of urban (black dots) and rural (white dots) breeding pairs at nests never occupied or occupied at least one year by a family unit. (**b**) Natal dispersal distances (in meters) of urban burrowing owls *Athene cunicularia* delaying dispersal (black points: observed values, black dashed line: median; n = 89 m) compared to the frequency distribution of distances expected if they were recruiting into the breeding population after dispersing during their first year of life (non-delayed dispersal).

Models also show that although the annual productivity of breeding pairs in nests occupied at least once by family units was higher than the productivity of breeding pairs in nests never occupied by a family unit (estimate: 0.10, 95% CI: −0.05—0.25), the main difference in this demographic parameter was linked to the habitat type (estimate for urban breeding pairs: 0.10, 95% CI: 0.05—0.15; Fig. 3a; see model fit in Fig. S3). Thus, while results are not conclusive, delayed dispersal and family unit formation seem to be more likely in high-quality nests.

### Consequences of delayed dispersal and family living

Individuals delaying dispersal and forming family units were observed recruiting into the breeding population as independent breeders only in urban areas, occupying a vacancy in their natal nest (two out of 14 individuals with known dispersal distances) or in its surroundings. Indeed, the median natal dispersal distance of individuals delaying dispersal (89 m, range: 0—413 m) is significantly lower (p < 0.01; Fig. 3b) than expected considering the natal dispersal distances of non-philopatric individuals that breed for their first time in their first year of life.

Our data do not show apparent fitness consequences for individuals adopting this family breeding strategy. The lifetime reproductive success of individuals delaying dispersal was almost the same as that of individuals independently breeding since their first year (mean LRS individuals delaying dispersal: 3.44 chicks, SD = 5.46, n = 9 individuals; mean LRS individuals non-delaying dispersal: 3.89 chicks, SD = 4.06, n = 145 individuals; Table 2). Regarding survival prospects, the 22 individuals delaying dispersal also show similar future apparent adult survival (mean = 0.75, 95% CI: 0.72–0.78) compared to those dispersing to breed independently in their first year of life (mean = 0.70, 95% CI: 0.58–0.82) (Z-test = 0.76, p = 0.22).

**Table 2:** Models obtained to assess differences in lifetime productivity of urban burrowing owls *Athene cunicularia* delaying dispersal and forming family units or breeding during their first year.

Model selection					Model averaging			
Model	k	AICc	∆AICc	w	Variable	Estimate	2.5% CI	97.5% CI
null	3	765.87	0.00	0.74	delayed dispersal	0.05	−0.77	0.87
delayed dispersal	4	767.97	2.10	0.26				

Estimates and 95% confidence intervals (2.5% and 97.5%) were assessed after model averaging. Models were corrected for zero-inflation. k: number of parameters, AICc: Akaike Information Criterion corrected for small sample sizes, ΔAICc: the difference between the AICc of model i and that of the best model (i.e. the model with the lowest AICc), w: Akaike weights. The fit of the model including all variables used in model averaging can be checked in Fig. S4.

When considering the consequences of delayed dispersal for adults accepting an extra-individual, we found that, despite habitat differences (i.e., higher productivity in urban than in rural nests), nests occupied by family units were more productive than those occupied by breeding pairs, an effect that was more marked among rural pairs (Table 3). However, when analyses were repeated considering only nests occupied at least once by a family unit, we did not find differences in productivity associated with the breeding unit (family or breeding pair) or the habitat type (Table 3), supporting the idea that delayed dispersal is more likely in high-quality nests. Conversely, the body condition of chicks was significantly higher in family units compared to breeding pairs, both when all nests and those occupied at least once by a family unit were considered. No differences between urban and rural habitats were detected in this case (Table 4). Thus, while individuals delaying dispersal do not increase the number of chicks fledged at a nest, they do have a positive effect on the body condition of these offspring.

**Table 3:** Models obtained to assess the effects of family units on the annual productivity of urban and rural burrowing owls *Athene cunicularia*.

Model selection					Model averaging			
Model	k	AICc	∆AICc	w	Variable	Estimate	2.5% CI	97.5% CI
**Models including all monitored nests**								
Habitat*family unit	6	16,884.34	0.00	0.73	**Habitat (urban)**	**0.10**	**0.06**	**0.15**
Habitat + family unit	5	16,886.75	2.41	0.22	**Family unit**	**0.27**	**0.10**	**0.44**
Habitat	4	16,889.79	5.45	0.05	Habitat (urban)*family unit	**−0.22**	**−0.42**	**−0.02**
Family unit	4	16,899.28	14.94	0.00				
Null	3	16,904.29	19.95	0.00				
**Models including only nests occupied at least once by a family unit**								
Null	4	2455.87	0.00	0.47	Habitat (urban)	−0.04	−0.17	0.09
Habitat	5	2457.48	1.61	0.21	Family unit	0.03	−0.09	0.15
Family unit	5	2457.64	1.77	0.20				
Habitat + family unit	6	2459.27	3.40	0.09				
Habitat*family unit	7	2461.17	5.30	0.03				

Estimates and 95% confidence intervals (2.5% and 97.5%) were assessed after model averaging. All models were run including year as a random term and were corrected for zero-inflation; models run to compare the productivity of family units and breeding pairs considering only the set of nests that were at least once occupied by a family unit also included “nest” as a random term. k: number of parameters, AICc: Akaike Information Criterion corrected for small sample sizes, ΔAICc: the difference between the AICc of model i and that of the best model (i.e. the model with the lowest AICc), w: Akaike weights. The fit of the models including all variables used in model averaging can be checked in Fig. S5. In bold, variables receiving strong support (i.e., the 95% confidence interval did not overlap with zero).

**Table 4:** Models obtained to assess the effects of family units on the body condition of chicks raised by urban and rural burrowing owls *Athene cunicularia*.

Model selection					Model averaging			
Model	k	AICc	∆AICc	w	Variable	Estimate	2.5% CI	97.5% CI
**Models including all monitored nests**								
Family unit	5	−5003.71	0.00	0.57	**Family unit**	**0.02**	**0.01**	**0.03**
Habitat + family unit	6	−5002.49	1.22	0.31	habitat(urban)	0.00	−0.01	0.01
Habitat*family unit	7	−5000.54	3.18	0.12				
Null	4	−4987.63	16.09	0.00				
Habitat	5	−4987.34	16.37	0.00				
**Models including only nests occupied at least once by a family unit**								
Family unit	5	−345.83	0.00	0.68	**Family unit**	**0.03**	**0.04**	**0.06**
Habitat + family unit	6	−343.60	2.23	0.22				
Habitat*family unit	7	−342.04	3.79	0.10				
Null	4	−326.15	19.68	0.00				
Habitat	5	−324.12	21.71	0.00				

Estimates and 95% confidence intervals (2.5% and 97.5%) were assessed after model averaging. All models were run including “year” and “nest” as random terms. k: number of parameters, AICc: Akaike Information Criterion corrected for small sample sizes, ΔAICc: the difference between the AICc of model i and that of the best model (i.e. the model with the lowest AICc), w: Akaike weights. The fit of the models including all variables used in model averaging can be checked in Fig. S6. In bold, variables receiving strong support (i.e., the 95% confidence interval did not overlap with zero).

**Figure 4:**
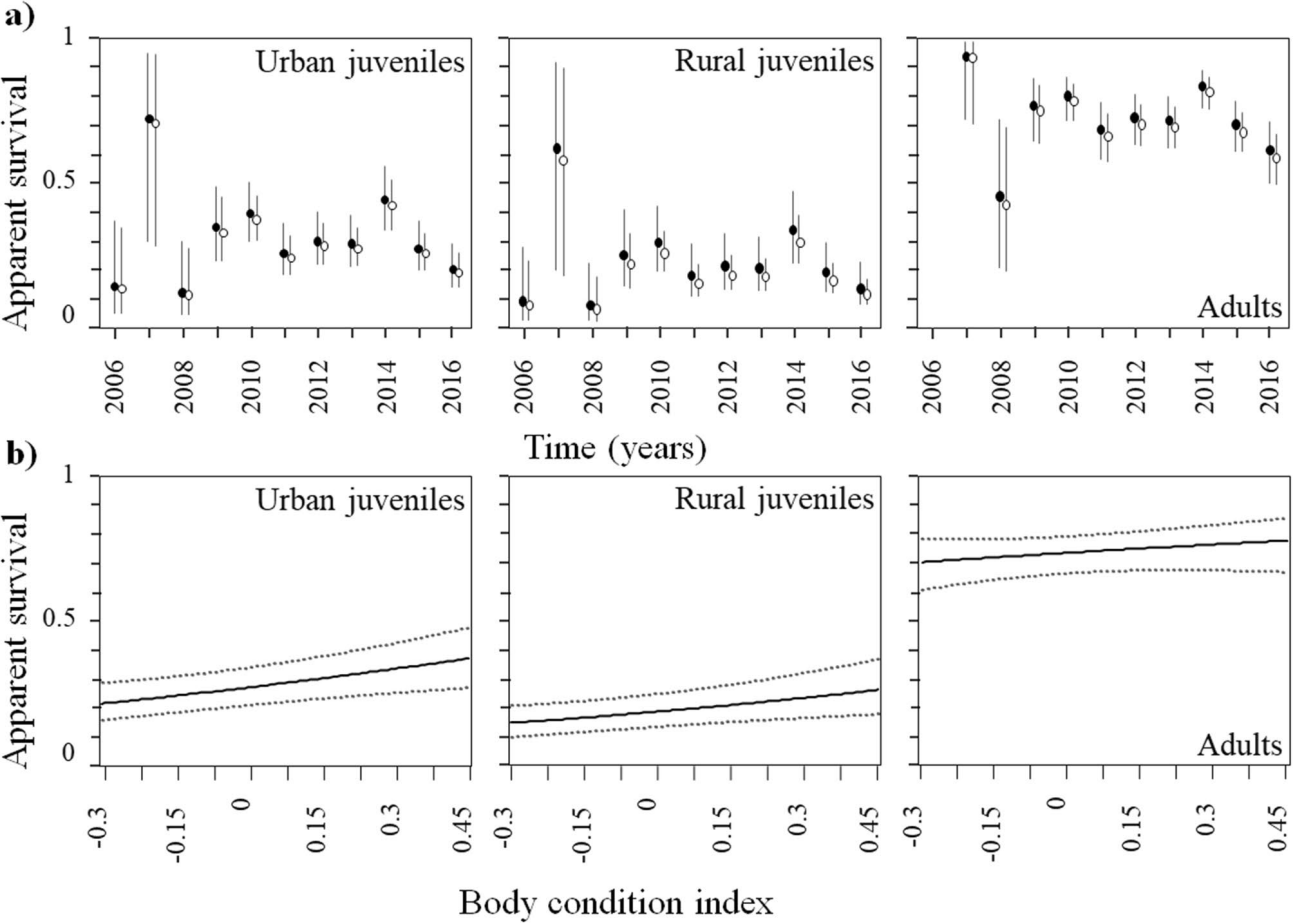
Model averaged (± 95% CI; bars and dotted lines) estimates of apparent survival probabilities for urban and rural juvenile and adult burrowing owls *Athene cunicularia* (**a**) raised in family units (black dots) and breeding pairs (white dots), and (**b**) in relation to individual body condition as chicks (we used the estimates for 2013 to plot the figures).

CMR models showed that the apparent future survival of individuals ringed as chicks changed along years, always being higher after reaching adulthood (Table 5, Fig. 4, Table S3). Moreover, while adult apparent survival was similar between habitats (mean apparent survival for adults: 0.71, 95% CI: 0.60—0.79), individuals born in urban habitats had a higher apparent juvenile survival than those of rural ones (across year mean apparent survival for urban juveniles: 0.29, 95% CI: 0.19—0.41; across year mean apparent survival for rural juveniles: 0.21, 95% CI: 0.12—0.31; Table 5; Fig. 4). Models including the effect of the breeding structure (i.e., family units vs breeding pairs) or not including this effect were close in terms of AICc (Table 5), and model-averaged estimates of future apparent juvenile and adult survival probabilities were similar for both groups (i.e., individuals born at family units or breeding pairs; Fig. 4a). On the other hand, future apparent survival was positively related to the body condition of chicks, with the model including the effect of body condition on both juvenile and adult survival being the best model in terms of AICc (estimate: 0.92, 95% CI: 0.42—1.42; Table 5). This result suggests that chicks in better body condition survived better than those in poorer condition (Fig. 4b).

**Table 5:** Effects of family units and body condition on the juvenile and adult survival probabilities of urban and rural burrowing owls *Athene cunicularia* ringed as chicks.

	k	AICc	ΔAICc	w
**Effect of family structure**				
(JuvU/JuvR/Ad) + time	15	2731.33	0	0.27
(JuvU/JuvR/Ad) + family unit + time	16	2731.74	0.41	0.22
(JuvU/(JuvR*family unit)/Ad) + time	16	2732.40	1.07	0.16
(JuvU/JuvR/(Ad*family unit)) + time	16	2732.41	1.08	0.16
((JuvU*family unit)/JuvR/Ad) + time	16	2733.13	1.80	0.11
(((JuvU /JuvR)*family unit)/Ad) + time	17	2734.20	2.86	0.06
((JuvU /JuvR /Ad) *family unit) + time	18	2735.42	4.09	0.03
**Effect of body condition**				
((JuvU/JuvR/Ad) + body condition) + time	16	2781.68	0.00	0.59
((JuvU/JuvR) + body condition)/Ad) + time	16	2782.68	1.00	0.36
((JuvU*body condition)/JuvR/Ad) + time	16	2787.38	5.70	0.03
(JuvU/(JuvR*body condition)/Ad) + time	16	2790.43	8.75	0.01
(JuvU/JuvR/(Ad*body condition)) + time	16	2791.94	10.26	0.00
(JuvU/JuvR/Ad) + time	15	2792.80	11.12	0.00

All models considered the effect of field effort on recapture probabilities (see Table S2). Note that for body condition analyses, all models considered differences between adults, urban juveniles, and rural juveniles and additive temporal variation among groups (see Table S3). k: number of parameters, AICc: Akaike Information Criterion corrected for small sample sizes, ΔAICc: the difference between the AICc of model i and that of the best model (i.e. the model with the lowest AICc), w: Akaike weights. + : additive effect, *: interactive effect. “/” indicates that different parameters exist for individuals from different classes. Brackets are used when a particular effect (e.g. time) is applied to different groups. JuvU: urban juveniles, JuvR: rural juveniles, Ad: adults.

Extra-individuals forming the family units rarely participate in nest defence (i.e., we only recorded an individual not forming the dominant breeding pair approaching or attacking the predator in one out of the 12 family units tested). Moreover, individuals forming family units took longer to approach (posterior mean: 0.60, 95% CI: 0.29—0.90) and performed less aggressive attacks toward the predator (number of aggressions: estimate: −2.33, 95% CI: −3.56 to −1.09) than individuals of breeding units, independently of habitat type (posterior mean for the minimum time to approach the predator in urban habitats: −0.04, 95% CI: −0.26 to 0.16; estimate for the number of aggressions in urban habitat: −0.24, 95% CI: −0.99 to 0.51). This pattern remained constant when we repeated the analysis considering the nest instead of the individual as a sample unit (minimum time to approach the predator: posterior mean: 0.36, 95% CI: 0.03—0.66, and total number of aggressions performed by all adults: estimate: −1.64, 95% CI: −2.94 to −0.34), not supporting the hypothesis that family units can provide benefits in terms of nest defence.

## Discussion

In many vertebrate species, sexually mature individuals can postpone natal dispersal, and thus reproduction, to remain as subordinates in family units, sometimes even helping dominant individuals to raise their offspring^61–63^. The costs and benefits of this strategy, as well as its drivers, have been investigated mainly in social species that usually bred cooperatively^64^. However, delayed dispersal and/or family living has also been described in non-social species^25,65^, suggesting that there may be a dynamic and taxonomically varied combination of factors influencing the evolution and maintenance of this strategy. Here, we show that the burrowing owl can form family units when individuals, mainly males, delay their dispersal to stay at their natal nests with one or both parents. This delayed dispersal, while rather infrequent, was more prevalent in urban habitats and in areas with high conspecific density and productivity. Although our sample size is small, birds delaying dispersal rarely contributed directly to the genetic pool of the offspring, did not contribute to nest defense, and did not increase the productivity of the nests where they remained. However, individuals delaying dispersal ultimately recruited into the breeding population occupying higher quality nests, closer to their natal nests than expected had they moved to search for a vacancy, unavailable in their natal area, to breed independently. It is worth mentioning that independent breeding after delayed dispersal was only detected in urban areas, never in rural ones, a fact that can explain why this strategy is even less common in the latter than in the former habitat. While we did not find direct fitness benefits in terms of increased lifetime reproductive success or apparent survival, perhaps due to our small sample size for delayed dispersers, the presence of individuals delaying dispersal in a nest was positively related to the body condition of the chicks raised, which was ultimately positively linked to offspring's future survival probabilities.

Dispersal can be postponed when suitable breeding sites are constrained (the ecological constraints hypothesis) mainly when habitats are saturated and gaining a breeding position elsewhere is, therefore, difficult^66–68^. Recent theoretical^12^ and empirical studies (e.g.^13,69–73^) have questioned the general role of habitat saturation in promoting delayed dispersal. However, offspring can also delay breeding and stay at their natal territories when the variability in habitat quality among breeding areas is so high that waiting for a good territory instead of attempting to breed in a low-quality vacancy or prospecting to obtain information about breeding opportunities elsewhere can provide benefits. When predation risk is high, as when individuals have to sample unfamiliar environments to find a suitable breeding option, dispersal can be particularly risky, so individuals can choose to stay in their natal territories^74,75^, which could serve as refuges^13,76^. High predation risk has thus been predicted to directly reduce the dispersal probability of individuals in social species, even favouring cooperative breeding^77^. Predation is the main determinant of breeding failure in burrowing owls^48,55^, also affecting juvenile survival (personal data), so individuals need to find ways to identify safe areas to establish themselves for breeding. Animals may use landscape features as a proxy of habitat quality^78,79^. However, when habitat quality is determined by predation risk and this risk is difficult to evaluate through landscape attributes, organisms may rely on indirect cues of intrinsic habitat quality, such as conspecific density^80–85^. Conspecific density can correlate with habitat quality due to the movement of individuals to high-quality patches and/or to the differential mortality of resident conspecifics. Thus, areas with a high density of conspecifics could represent areas where predation risk is rather low^48^. In our study system, urban areas in general, and rural areas with a high density of conspecifics can represent safe spaces where individuals can expect to maximise their long-term fitness. Previous results show that burrowing owls born or breeding in areas with a high density of conspecifics tend to remain closer (lower natal and breeding dispersal distances) than those in low-density ones^55,56^. Moreover, natal dispersal distances in our study species are lower for males than for females^56^ due to benefits from remaining near a known area, where they are most familiar with resources and are probably best able to compete for them^86–88^. Indeed, offspring remained in their natal nests far beyond their independence, constraining their opportunities of prospecting for vacant territories to the surrounding of these areas. However, intraspecific competition or a lack of mates can preclude some males from recruit into a breeding site close to their natal areas in those high-quality areas. Thus, delaying dispersal, without costs in terms of lifetime productivity and survival, and gaining the opportunity to inherit a good quality vacancy^11,29,89^, could be the best choice for some males.

Predation can lead to delay in dispersal, but it could also preclude it by reducing the probability that a breeder remains alive in the followed breeding season. Parents, through passive tolerance or active investment^31^, are critical in allowing delayed dispersal, to the point that several studies have shown that their replacement by a stepparent (one, mainly the male, or both of them) is associated with a higher likelihood of offspring dispersal^90,91^. In the burrowing owl, however, this may not happen. Our data show that in six out of the 14 genotyped families, the breeding male was not the father of the individuals delaying dispersal. The acceptance of non-direct offspring can be related to the fact that adult burrowing owls do not actively provide food or differentially defend their retained offsprings from predators as in other species^92,93^. However, their passive tolerance toward individuals remaining in the territory allows these non-breeding birds to gain access to food in high quality areas and benefit of the antipredator protection of the main breeders.

The consequences of delayed dispersal on the fitness components of individuals forming family units are difficult to assess^94^. Here, we found that burrowing owls delaying dispersal may benefit from recruiting into high-quality nests close to their natal areas (the “safe-haven” hypothesis^13^) without paying costs in terms of long-term productivity or apparent survival. These latter results, however, should be considered with caution, as sample sizes were too low to support strong conclusions. Nevertheless, the presence of these extra-individuals has a positive effect on the body condition of the offspring raised in those nests, which ultimately affects the future survival of the brood, actually improving the reproductive output of the dominant breeding pair and their inclusive fitness. These extra-individuals did not actively participate in or increase the effectiveness of nest defence (indeed, individuals in family units took longer to approach the predator and performed fewer aggressions toward it than breeding pairs), but may help during offspring food provisioning. Unfortunatively, we have not recorded the contribution of different family members on offspring provisioning, a key aspect that merits further research.

Previous studies exploring factors affecting the decision of an individual to delay dispersal focused on social species, where results are strongly affected by the intrinsic benefits of group living and kin selection^64,95,96^. Using a non-social species living in habitats exhibiting contrasting ecological pressures, we found evidence supporting how the benefits of philopatry, combined with the heterogeneity in habitat quality, can promote delayed dispersal. Although there are no associated costs for the individual delaying dispersal nor the dominant individuals of the breeding territory, the low rewards in terms of individual’s fitness and the high turnover rate at territories can explain its low frequency and occurrence only in particular ecological situations^25,97^. This study contributes to understanding the role played by habitat characteristics, mainly those related to predation risk, in promoting delayed dispersal and family living not only in social but also non-social species, highlighting its impact on the ecology and evolution of animal populations.

## Material and methods

### Study species and area

The burrowing owl is a small, long-legged owl found across open landscapes of North and South America (i.e., grasslands, rangelands, agricultural areas, and deserts) that nests and roosts in burrows excavated by themselves or by mammals^98^. Burrowing owls are highly conspicuous in the daytime during the breeding season, and thus easily located usually within 30 m of their nests. In the Northern Hemisphere, the transformation of grasslands and the introduction of contaminants into the environment seem to be leading negative trends in migratory populations^99^. In South America, however, where the species is a year-round resident, it is relatively common in areas with different levels of grazing pressure^48^ and, in recent years, in urban environments^41^. Although individuals belonging to the same family can roost together near their burrow even after offspring are independent and some clusters of breeding pairs can be observed in the field, pairs are territorial^100^ and individuals do not perform collective nest defence (except the member of the same breeding pair) or foraging, so they cannot be considered as a typical social species. The mean lifespan of burrowing owls is relatively short, ranging between 1.3 and 2.9 yrs^57,101^. Individuals typically recruit into the breeding population during their first year of life in September–October^56^. Our long-term monitoring (from 2006 to 2018) suggests that there are no floaters or, at least, that this strategy is extremely rare.

Our study area encompasses 5,400 km^2^ of large rural expanses of natural and transformed grasslands around Bahia Blanca city (Buenos Aires, Argentina). Rural owls breed in natural grasslands and pastures dedicated to cattle raising where human presence is rare and mostly restricted to some scarce roads and scattered farms^102^. Urban owls, conversely, excavate their nests in private gardens, public parks, unbuilt spaces among houses, roundabouts, and large avenues, in continuous contact with people and traffic. The city is immediately surrounded by large rural expanses of natural and transformed grasslands, without barriers that may constrain the movements of owls between habitats^101^. Moreover, as owls can excavate their burrows, their dispersal is not expected to be constrained by territory availability^36,56^.

From 2006 to 2018, we monitored the breeding populations of the species in the study area anuually, totalling ca. 2,900 urban and 3,500 rural nests during the whole period. Breeding sites were repeatedly visited from November to January to determine the identity of the breeding individuals, their productivity (i.e. the number of young fledged per breeding attempt), and to capture adults and chicks using bow nets and ribbon carpets. In the breeding season of 2009–2010, we also selected 76 urban and 62 rural nests with fledgings (n = 353) to monitor the time during which these individuals remained in their natal nests with their parents. These nests were visited twice per month -from January to July- to record the presence of fledgings and their identity (when individuals were ringed).

All captured birds were marked using plastic rings with an individual alphanumeric code and released after measuring (wing length, in millimetres), weighing (in grams), and bleeding (0.1 ml) them. Blood samples were preserved in absolute ethanol and kept at 4 °C until their processing in the laboratory. Individuals were sexed based on plumage characteristics^101^ and, when needed, by molecular procedures^36^.

### Delayed dispersal as a route to family living

The monthly percentage of young individuals that remained in their natal nests was compared between urban and rural habitats using Generalized Linear Models (GLM; binomial error distribution, logit link function). Models included time, habitat, and their interaction. To avoid potential errors associated with the potential movement of individuals among nests, the same analysis was repeated using only birds ringed as chicks.

Only 340 individuals out of the 1,579 birds ringed as chicks were resighted during their first breeding attempt, most of them (318 individuals) forming breeding pairs (one male and one female) at different distances to their natal areas^56^. However, ca. 7% of these individuals (n = 22) stayed at their natal nests the following breeding season, delaying dispersal and forming family units. Previous studies have confirmed genetic monogamy in the burrowing owl^36^, so the relatedness among the components of family units can be assessed using resighting data of individuals marked as chicks of known parents. As the sample size obtained through this procedure is low, we captured and genotyped using a panel of 17 polymorphic microsatellites previously tested for the species (see^36^ for details), 60 individuals belonging to 14 family units, encompassing five complete families (all the adults plus their chicks), four family units (all the adults) with no chicks and five that lacked the mother's genotype. This same procedure allowed us to ascertain the genetic contribution of the three members of the family units to their brood.

DNA was extracted from blood samples using a modification of the silica-based protocol (105). Briefly, all loci were PCR amplified in two independent multiplex reactions^36^. Genotypes were assigned, both manually and automatically, using GeneMapper 3.7 (Applied Biosystems, Foster City, CA), and all electropherograms were double-checked independently by two people. All microsatellites were at Hardy–Weinberg and linkage equilibrium^36^. We calculated maximum likelihood relatedness to determine the most probable kin relationship among individuals using ML-Relate software^103^ and performed parentage analyses on every chick to assess the genetic contribution of the members of the family units using the program Cervus 3.0.3^104^. Cervus applies a likelihood-based approach to assign parentage combined with simulation of parentage analysis to determine the confidence of parentage assignments. We generated 100,000 simulated offspring, assuming 0.7% sampled parents, 99% loci typified, an inbreeding rate of 0.06%, and a genotyping error of 0.01^36^.

### Factors driving delayed dispersal and family units

We used Generalised Linear Mixed Models (GLMM) to investigate factors promoting delayed dispersal and, thus, the formation of family units in our study populations. To achieve this goal, we modelled the probability of a nest being occupied by a breeding pair or a family unit (binomial error distribution, logistic link function, n = 5776 breeding events), including as explanatory variables conspecific density and productivity (social variables) and the habitat type (urban or rural), as well as their interactions. “Year” was considered as a random term in models to control for potential interannual differences. We did not include “nest” as a random term due to convergence problems because only 35% of the nests were repeatedly used by owls through the study period. Conspecific density was calculated using an annual aggregation index for each breeding site, obtained as their relative position within the spatial distribution of all breeding sites^25^. This index was obtained using the GPS nest location of all breeding nests as *Si* = *Σ exp* (− *d*_*ij*_) (with *i* ≠ *j*), where *d*_*ij*_ was the linear distance between nest *i* and *j*. We also estimated conspecific productivity in the surroundings of each breeding site using a modification of this aggregation index, where the distance to each breeding pair was weighted by its productivity. Conspecific productivity was then obtained as the residual of this last variable against the aggregation index calculated previously^56^. All covariates were centred before modelling to properly estimate their main effects^105^.

Complementarily, we assessed whether delayed dispersal, and thus family living, was more likely in high-quality nests by comparing, also using GLMM, the productivity (Poisson error distribution, log link) of breeding pairs in nests occupied at least once by family units, and in nests never occupied by a family unit (sample sizes: urban nests never occupied by family units or occupied at least once: 1150 and 106 breeding sites, respectively; rural nests never occupied by family units or occupied at least once: 1973 and 45 breeding sites, respectively). Models were built that included habitat, the occupation of the nest by a family unit at least once and their interaction as fixed terms, and “year” and “nest” as random effects.

### Consequences of delayed dispersal and family living

We considered potential benefits of delayed dispersal, and thus family living, for individuals adopting this strategy as well as for the dominant individuals of the nest where they remained and their offspring. We focused on individuals delaying dispersal to, firstly, assess whether they benefit by later recruiting into the breeding population at distances closer to their natal nests than those breeding independently from their first year of life. Towards that end, we compared the observed natal dispersal distance of individuals delaying dispersal (the linear distance between the natal nest and the nest where the individual recruit as a breeder in its second year of life; n = 14) with the expected distribution obtained after shuffling the natal dispersal distances recorded for non-philopatric individuals. Philopatric birds were excluded from this analysis because we consider delayed dispersal as an alternative strategy to dispersal when individuals cannot independently breed in their natal territory (philopatry) due to the lack of vacancies. We only focussed on urban birds (n = 208) because we did not record rural individuals delaying dispersal that later successfully recruited into the breeding population. The significance test was generated by counting the number of randomised cases that resulted in an equal or smaller value to the observed dispersal distance and then divided by 1000 (i.e. the total number of randomisations).

Second, we used GLM to compare lifetime reproductive success of individuals (log link function, negative binomial error distributions) delaying dispersal (n = 9 urban individuals) or breeding during their first year (n = 145 urban individuals). Lifetime reproductive success (i.e., the total progeny an organism can produce in their lifetime) was calculated considering individuals ringed as chicks with known reproductive output for every year during their reproductive careers and not seen during at least two years before the end of this study (until 2016), which had a very high probability of being dead (probability of not resighting a living individual over two years was < 0.04^56^). For individuals delaying dispersal, we also included in the estimate of lifetime productivity the number of chicks raised during the year when they remained in their natal nest, assuming that the coefficient of relatedness for first-degree relatives (i.e., parents and offspring or full siblings) is the same^106^. Models included the dispersal strategy of individuals (delayed dispersal or dispersal) as a fixed factor.

Third, we compared the future survival probabilities of 1-year-old individuals delaying or not delaying dispersal. We selected the 22 encounter histories of individuals delaying dispersal and forming family units and we randomly selected 22 encounter histories of non-philopatric individuals (see before) not delaying dispersal in identical proportions to their natal cohort. We generated 200 datasets with randomly selected encounter histories and we estimated survival probabilities for both groups (considering a constant recapture parameter) using the program R-MARK^107^. We compared the difference in survival estimates for individuals delaying dispersal and individuals not delaying dispersal using a Z-test^108^.

We then assessed the consequences of delayed dispersal for the dominant individuals of the nests where individuals stay by comparing productivity, offspring body condition, and antipredatory behaviour (see below) in nests occupied by breeding pairs and family units. To do so, we first used GLMM to compare annual productivity (Poisson error distribution, log link) of urban and rural breeding pairs and family units (sample sizes: urban family units and breeding pairs: 167 and 2150 breeding events, respectively; rural family units and breeding pairs: 78 and 2653 breeding events, respectively), including habitat as fixed factors and year as random terms in models (as before, convergence problems prevented the inclusion of nest as a random term). As delayed dispersal can be more likely in high-quality nests (see above), we also compared using GLMM the productivity (Poisson error distribution, log link) of breeding pairs and family units in nests occupied at least once by a family unit (sample sizes: urban family units and breeding pairs: 167 and 322 breeding events, respectively; rural family units and breeding pairs: 78 and 107 breeding events, respectively). Models included habitat, the presence of a family unit, and their interaction as fixed terms and “nest” and “year” as random effects.

To evaluate the effects of family units on offspring body condition, we used two complementary approaches. On the one side, we used GLMM to compare body condition of chicks (normal error distribution, identity link function) born in breeding pairs and family units, and included habitat as a factor in models. As before, family units can be more likely in high-quality nests, so we used alternatively all monitored nests as well as nests occupied at least once by a family unit. The body condition of chicks was estimated as the residuals of a log–log regression of body mass on wing length (1.45 + 0.37*(log)wing length^109^). Models included “nest” and “year” as random effects to control for potential non-independence in data and interannual differences. We then used capture-mark-recapture models (CMR) to assess whether individual survival was related to natal conditions, mainly body condition and the breeding structure where they were raised (breeding pairs or family units). We used encounter histories of 1,407 individuals marked as chicks (2,060 resightings), subsampling 1,397 individuals with information on body condition. We started our modelling procedure by considering the interaction among time, habitat (urban or rural), and age (juvenile or adult) on survival, and testing the effects of time, habitat, and fieldwork effort on recapture. The variable fieldwork effort was created to differentiate years of low (2007 and 2008) and high monitoring effort (2009 to 2017). Once the best structure for recapture was selected, we modelled the effects of habitat and time on survival. Using the best survival and recapture structures, we separately tested the effect of family units and breeding pairs, and the effect of individual body condition on juvenile and adult survival of the offspring. We evaluated the goodness-of-fit (GOF) of the Cormack-Jolly-Seber model including two age classes and habitat and breeding structure effects (i.e. groups) using the program U-CARE^110^. The overall GOF was not statistically significant, thereby indicating a good fit to the data (χ^2^ = 34.34, df = 43, p = 0.824). CMR analyses were carried out using the program E-SURGE 2.1.4^111^ but the specific effect of individual covariates (i.e. body condition) on the reduced dataset was tested using the program MARK^112^.

Finally, we experimentally evaluated whether the antipredatory behaviour of family units differed from that of breeding pairs. For this purpose, we placed a polyester reproduction of a native predator, the Pampa Fox (*Pseudalopex gymnocercus*), close to the entrance (1 m) of the nests for 15 min to minimise disturbance^52^. We tested antipredatory behaviour in 42 urban and 36 rural pairs and in three rural and nine urban family units. To homogenise the underlying state of the individuals as much as possible, experiments were only performed in nests where adults were rearing medium-aged chicks, excluding those with fledglings or unsuccessful nests. During these simulated predation events, we measured the minimum time (in minutes) that each individual took to approach the predator (i.e., when the individual perched close to the predator, performed displays, and was ready to attack), and the total number of aggressions toward the artificial fox. We recorded observations from a distance using binoculars and telescopes to avoid interfering with the activity of owls. Subsequent visits to the nests confirmed that there were no alterations in the behaviour of the adults toward their offspring or nest failures associated with the experiment. We used a Bayesian Markov chain Monte Carlo technique implemented in the MCMCglmm package in R^113^ to model the latency to approach a predator (log-transformed to reach normality) as a dependent variable, including family structure and habitat as a fixed effect and nest as a random term. Models were run with priors for the random variances set to 1, and a degree of belief n = 2. We used a “cengaussian” distribution as latencies were right-censored. Estimates were insensitive to the choice of priors (prior variances range 0.01–100). Parameter expansion was used to avoid poor mixing if variance component estimates were close to zero. All models were run for 100,000 iterations, preceded by a burn-in of 10,000 iterations. Estimates of parameters were stored every 25th iteration to reduce autocorrelation.

### Model fit and selection

All GLM and GLMM were fitted using the package glmmTBM, including a zero-inflation component when needed^114^. We used the package DHARMa^115^ to evaluate the fit of the models. DHARMa employed a simulation-based approach to create standardized residuals (values between 0 and 1) for fitted (generalized) linear (mixed) models and test the significance of the dispersion parameter, zero-inflation, and goodness-of-fit of the model (H_0_: fitted model suits well for the data).

GLM, GLMM, and CMR model selection were performed using the Akaike Information Criterion corrected for small sample sizes, AICc^116^. Within each set of models (which includes the null model but not models that did not converge), we calculated the ΔAICc (as the difference between the AICc of model i and that of the best model) and the Akaike weight (w) of each model. Models within 2 AICc units of the best one were considered as alternatives and, when needed, used to perform model averaging (package MuMIn^117^). We considered that a given effect received no, weak, or strong support when the 95% confidence interval (CI) strongly overlapped zero, barely overlapped zero, or did not overlap zero, respectively. For MCMCglmm, we tested the statistical support of the fixed effect by evaluating whether the posterior distributions of variables included in models (95% credible interval) overlapped (or not) zero. We did not use DIC to compare models as its application to model averaging is not well implemented in widely used statistical packages.

### Ethics statements

Fieldwork and procedures were conducted under permits from the Argentinean wildlife agency (22,500–4102/09), and the owners of private properties, in accordance with the approved guidelines of the Consejo Superior de Investigaciones Científicas CSIC (CEBA-EBD-11-28). This study was approved by the Ethic Committee of the Consejo Superior de Investigaciones Científicas CSIC.

## Electronic supplementary material

Below is the link to the electronic supplementary material.

Supplementary Information 1
