# Supplementary material for: Urban life promotes delayed dispersal and family living in a non-social bird species
Source: Sci Rep. 2021 Jan 8;11:107. doi: 10.1038/s41598-020-80344-8 (PMC7794495; doi:10.1038/s41598-020-80344-8)
Supplement: Supplementary file 1 — Supplementary Information 1 [file 41598_2020_80344_MOESM1_ESM.docx]

**SUPPLEMENTARY MATERIAL**

**Urban life promotes delayed dispersal and family living in a non-social bird species**

**Álvaro Luna, Nicolás A. Lois, Sol Rodríguez-Martinez, Antonio Palma, Ana Sanz-Aguilar, José L. Tella & Martina Carrete**

**1. Genetic analysis**

**Table S1.** Parentage analysis performed using programs Cervus and ML-Relate. The putative father of the chicks is indicated in bold. Superscripts: relationship between the main breeders (male 1 and female) and the individual delaying dispersal (male 2): M (Mother), F (Father), FS (Full-sibling), HS (Half-sibling), U (Unrelated).

| year | Territory | Family unit | | |  | Offspring | | |
| --- | --- | --- | --- | --- | --- | --- | --- | --- |
|  |  | Male 1 | Female | Male 2 |  | Chick 1 | Chick 2 | Chick 3 |
| 2006 | 1^1^ | BRJ4^F^ | BRJ7^U^ | BRJ5 |  | B6J | BRJV |  |
| 2009 | 2^1^ | **RA6**^F^ | BC2^M^ | R2K |  | RAU | R2A |  |
| 2011 | 3 | **R5E**^HS^ | A56^U^ | RFU |  | A4H | A13 |  |
| 2012 | 4 | **A70**^F^ | AJL^FS^ | AML |  | A8M^3^ | A6V | AX2 |
| 2012 | 5^2^ | AJU^FS^ | A7N^M^ | R4K |  | A89 | A69 |  |
| 2011 | 11 | RRM^F^ | RTJ^U^ | A00 |  |  |  |  |
| 2010 | 12 | R0K^HS^ | R0B^U^ | R11 |  |  |  |  |
| 2012 | 13 | R07^FS^ | AM9^M^ | RL5 |  |  |  |  |
| 2009 | 6 | **BC1**^U^ | - | R0G |  | R3D | R3E |  |
| 2010 | 7 | **RCR**^U^ | - | RZW |  | RRV | RJU |  |
| 2011 | 8 | **R0K**^HS^ | - | R11 |  | A32 | A2P | A0Y |
| 2011 | 9 | **RL5**^FS^ | - | R07 |  | A33 | A1A |  |
| 2012 | 10 | A36^F^ | - | **AL0** |  | A7U | A8A | AJT |
| 2011 | 14 | RR9^HS^ | - | AL8 |  |  |  |  |

^1^ High inbreeding between potential parents, inconclusive paternity analyses results.

^2^ High mismatch (~6-8) between mother and chicks (Potential intra-brood parasitism)

^3^ Unrelated to female in the nest, but son of male 1.

**2. Model fits**

Diagnostic tools provided by the DHARMa package in R (Hartig 2018) were used to evaluate the fit of the final models. DHARMa simulates quantile residuals from a fitted GLM or GLMM that are standardized to values between 0 and 1. For a correctly specified model, these residuals should have a uniform distribution regardless of the underlying model structure and can be interpreted similarly to residuals for linear models. The package includes statistical tests on the residuals to check for uniformity, overdispersion and zero inflation.

**Figure S1.** Non-parametric dispersion test and standard residuals plots for the final models obtained to describe the monthly percentage of young individuals that remained in their natal nests in urban and rural habitats. **a)** Model built using all monitored offsprings; **b)** Model built using only ringed offsprings. No significant problems were detected.

**
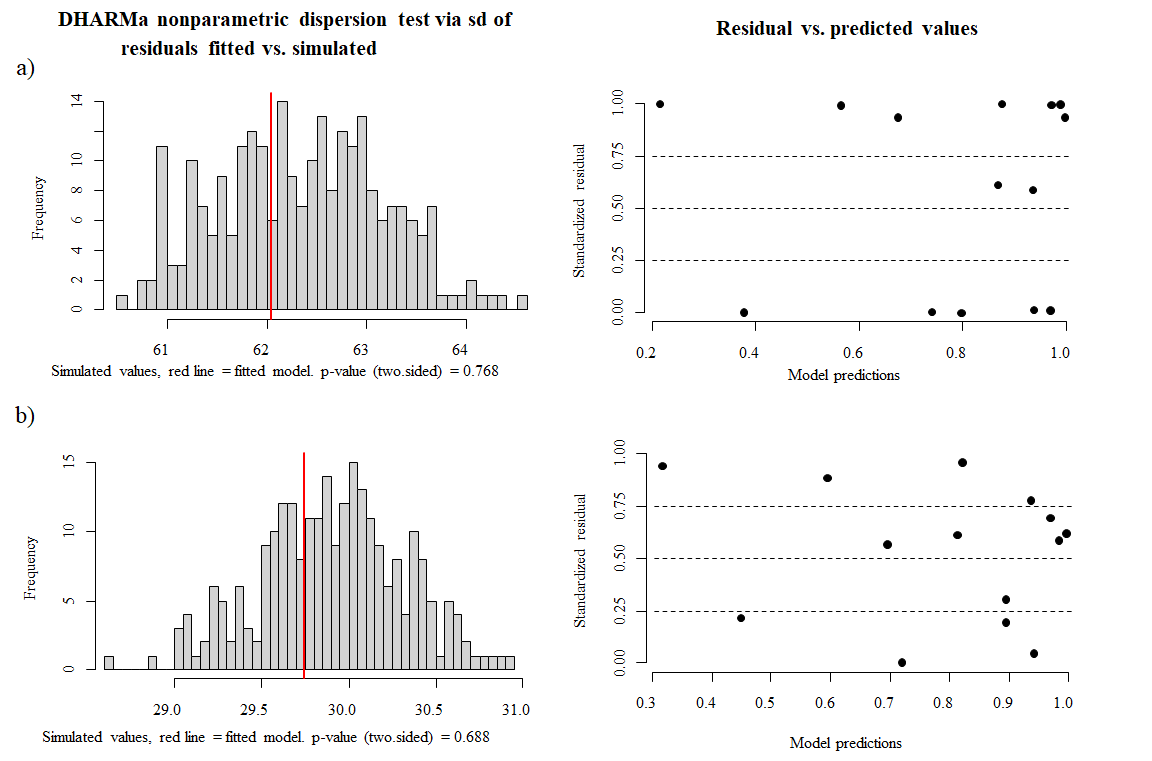
**

**Figure S2.** Non-parametric dispersion test and standard residuals plots for the final model obtained to assess the effects of habitat, conspecific density, and productivity on the probability of a nest being occupied by a family unit of burrowing owls. No significant problems were detected.

**
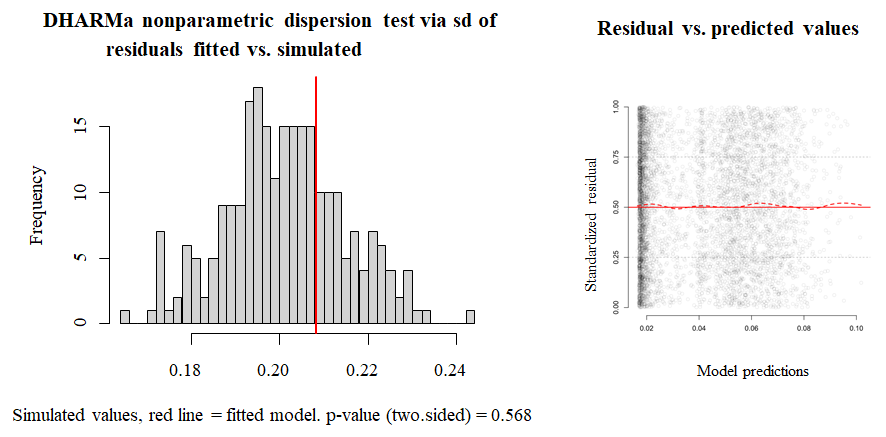
**

**Figure S3.** Non-parametric dispersion test, qq-plot, and standard residuals plots for the final model obtained to compare the annual productivity of breeding pairs in nests occupied at least once by family units or never occupied by a family unit of burrowing owls. Although the dispersion test was significant (0.94, p < 0.001), the qq-plot does not detect serious overall deviations from the expected distribution, so we did not consider that the model has significant problems that invalidate it.**
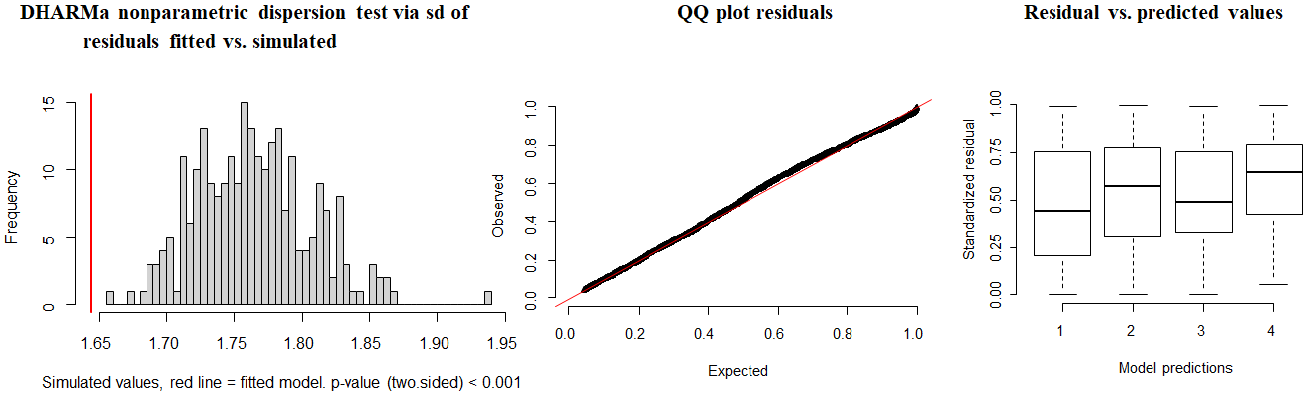
**

**Figure S4.** Non-parametric dispersion test and standard residuals plots for the model obtained to assess differences in lifetime reproductive success between individuals delaying or not delaying dispersal. No significant problems were detected.

**
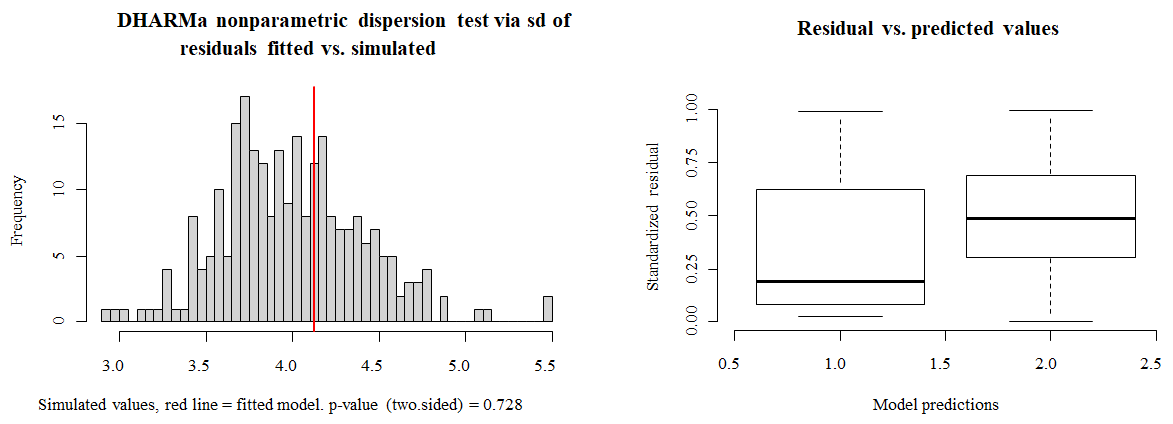
**

**Figure S5.** Non-parametric dispersion test, qq-plot, and standard residuals plots for the final model obtained to assess the consequences of delayed dispersal for adults accepting an extra-individual considering all nests (a) and only those occupied at least once by a family unit (b). Although the dispersion tests were significant (0.93 and 0.92, all-p < 0.001, respectively), the qq-plots do not detect serious overall deviations from the expected distribution, so we did not consider that the models have significant problems that invalidate them.

**
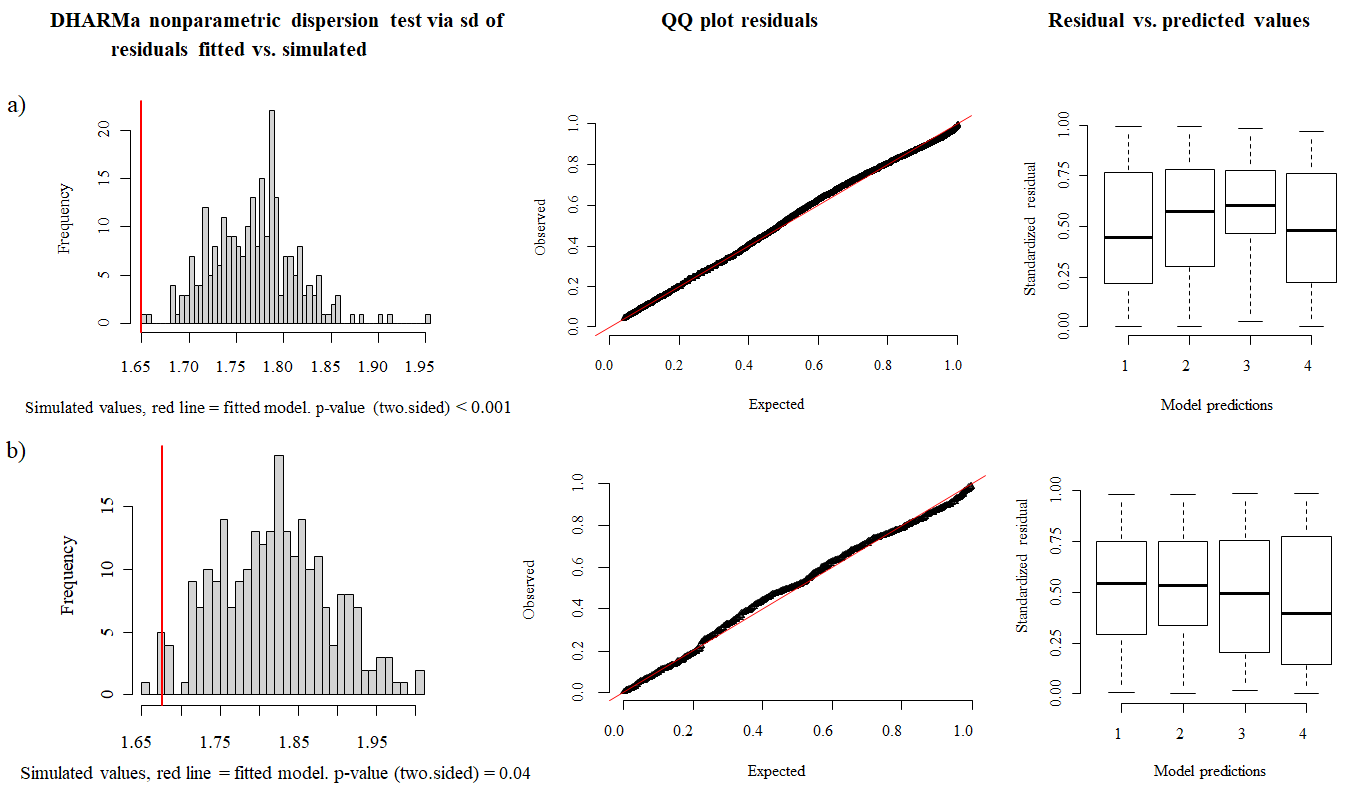
**

**Figure S6.** Non-parametric dispersion test and standard residuals plots for the model obtained to assess differences in the body condition of chicks in nests occupied by a breeding pair or a family unit (a) including all monitored nests or (b) only nests at least occupied once by a family unit. No significant problems were detected.

**
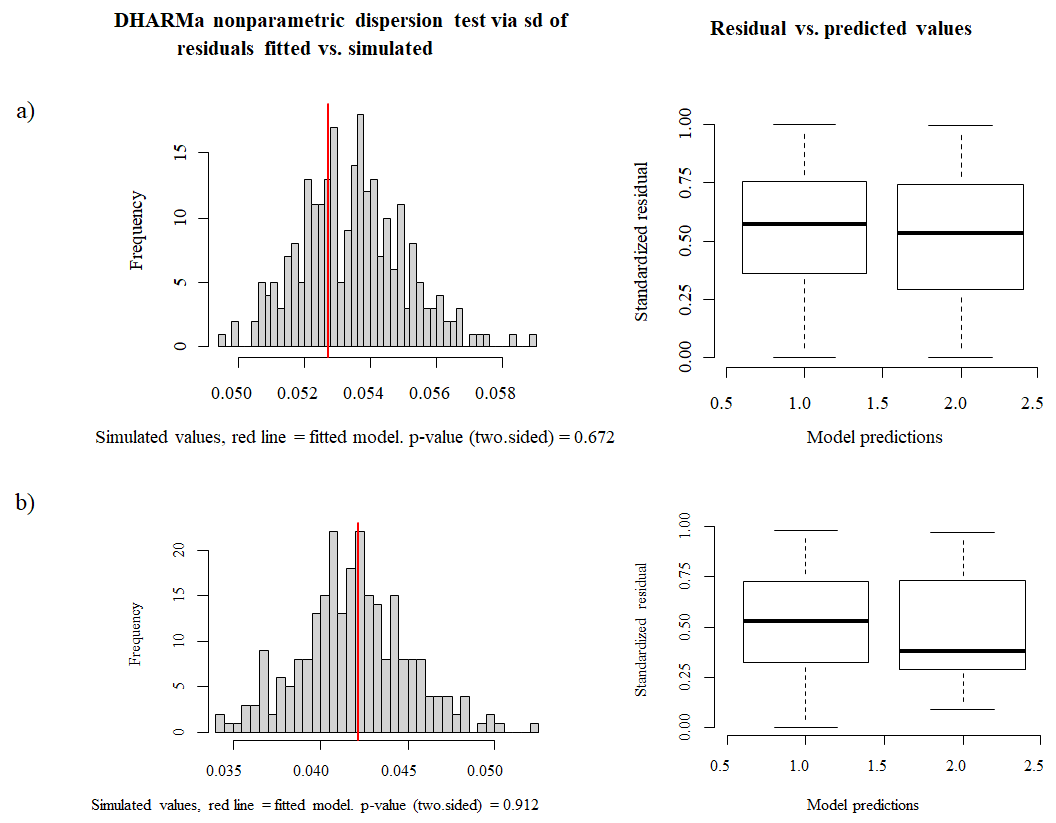
**

**3. Capture-Mark-Recapture Models**

We used capture-mark-recapture models (CMR) to assess whether juvenile or adult survival of chicks was related to their body condition and to the natal breeding structure where they were raised (breeding pairs or family units). We started our modelling procedure by considering the interaction among time, habitat (urban or rural), and age (juvenile or adult) on survival, and testing the effects of time, habitat, and fieldwork effort on recapture. The variable fieldwork effort was created to differentiate years of low (2007 and 2008) and high monitoring effort (2009 to 2017).

**Table S2.** Modelling the effects of habitat (urban and rural), fieldwork effort “effort”, and time on recapture probabilities. k: number of parameters estimated by the model age: two age classes differentiating juveniles and adults. . “+” indicate additive effects. “*” indicate interaction.

| **Survival** | **Recapture** | **K** | **deviance** | **AICc** | **ΔAICC** |
| --- | --- | --- | --- | --- | --- |
| age*habitat*time | effort | 44 | 2741.88 | 2751.99 | 0 |
| age*habitat*time | constant | 43 | 2745.34 | 2753.29 | 1.27 |
| age*habitat*time | habitat+effort | 45 | 2741.82 | 2754.01 | 2.03 |
| age*habitat*time | habitat | 44 | 2745.32 | 2755.33 | 3.34 |
| age*habitat*time | time | 51 | 2732.77 | 2757.82 | 5.83 |
| age*habitat*time | habitat+time | 52 | 2732.65 | 2759.8 | 7.82 |

Once the best structure for recapture was selected, we modelled the effects of habitat and time on survival. Using the best survival and recapture structures, we separately tested the effect of family units (or breeding pairs) and the effect of individual body condition on juvenile and adult survival of the offspring.

**Table S3.** Modelling the effects of age (juvenile “Juv” and adults “Ad”), habitat (urban “U” and rural “R”), and time on survival probabilities. k: number of parameters estimated by the model, effort: fieldwork effort. “/” indicates that different parameters exist for individuals from different classes. Brackets are used when a particular effect (e.g. time) is applied to different groups. “+” indicates additive effects. “*” indicates interaction.

| **Survival** | **Recapture** | **k** | **deviance** | **AICc** | **ΔAICC** |
| --- | --- | --- | --- | --- | --- |
| (JuvU/JuvR/Ad)+time | effort | 15 | 2782.13 | 2731.33 | 0.00 |
| (Juv/JR/AdU/AdR)+time | effort | 16 | 2782.03 | 2733.26 | 1.93 |
| (Juv/Ad)+habitat+time | effort | 15 | 2784.99 | 2734.11 | 2.77 |
| (Juv/Ad)+time | effort | 14 | 2794.60 | 2741.41 | 10.08 |
| (AdU/AdR/Juv)+time | effort | 15 | 2794.08 | 2742.94 | 11.60 |
| (Juv/Ad)*time | effort | 23 | 2779.36 | 2744.95 | 13.62 |
| (JuvU/JuvR/AdU/AdR)*time | effort | 44 | 2741.88 | 2751.99 | 20.66 |
| (JuvU/JuvR/AdU/AdR) | effort | 6 | 2827.68 | 2757.36 | 26.03 |
| (Juv/Ad)+habitat | effort | 5 | 2831.25 | 2758.81 | 27.48 |
| (Juv/Ad) | effort | 4 | 2843.86 | 2769.05 | 37.71 |
